# Incidence and progression of trachomatous scarring in a cohort of children in a formerly hyper-endemic district of Tanzania

**DOI:** 10.1371/journal.pntd.0008708

**Published:** 2020-10-05

**Authors:** Michael Saheb Kashaf, Beatriz E. Muñoz, Harran Mkocha, Meraf A. Wolle, Fahd Naufal, Sheila K. West

**Affiliations:** 1 Dana Center for Preventive Ophthalmology, Wilmer Eye Institute, Johns Hopkins University, Baltimore, MD, United States; 2 Kongwa Trachoma Project, Kongwa, United Republic of Tanzania; Ben-Gurion University of the Negev, ISRAEL

## Abstract

**Background:**

Trachoma is the leading infectious cause of blindness. Repeated or persistent ocular infection with *Chlamydia trachomatis* in childhood leads to conjunctival scarring, usually in adulthood but often earlier in areas with greater disease burden. There are limited longitudinal data examining change in scarring in children, especially where trachoma rates are low.

**Methodology/Principal findings:**

A cohort of children, ages 1–9 years, were randomly selected at baseline from 38 communities in Kongwa, Tanzania and followed for 2 years. Rates of trachomatous inflammation—follicular (TF) were <5% over the survey period. At baseline, 1,496 children were recruited and 1,266 (85%) were followed-up. Photographs were obtained at baseline and follow-up and graded for the presence and severity of scarring using a four-point scale ranging between S1-S4. In children without scarring at baseline, 1.6% (20/1,246) were found to have incident scarring, and incident scarring was more common among girls compared to boys. Among children with scarring at baseline, 21% (4/19) demonstrated progression.

**Conclusions/Significance:**

In this formerly hyper-endemic district, the incidence of new scarring in children ages 1–9 years is low, although 21% of those who had scarring at baseline progressed in severity over the 2-year follow-up period. These data provide support for the thesis that while incident scarring more closely reflects ongoing exposure, progression may involve factors independent of ongoing transmission of trachoma.

## Author summary

Trachoma is the leading infectious cause of blindness. The disease is caused by repeated eye infection with the bacterium *Chlamydia trachomatis* and characterized clinically in its active stage by follicles (TF) and/or severe inflammation (TI). Scarring of the inner surface of the eyelid results from repeated exposure to infection during childhood, and while active trachoma is mostly observed in childhood, scarring and late complications are often only seen in adulthood. In areas with heavy burden of trachoma, scarring may be observed among children, particularly among those with constant, severe trachoma and/or infection. Few studies have examined the incidence and progression of scarring in children in areas where the prevalence of trachoma has been substantially reduced. In this study, we examined the 2-year incidence and progression of scarring in Kongwa district, Tanzania where the prevalence of TF was about 5%. Incidence of new scarring was 0.8%/year and more common among girls. Progression of scarring was 21% in our cohort of children ages 1–9 years. Incidence in children was low in this formerly hyper-endemic area, but a substantial fraction of those with scarring at baseline demonstrated progression over the follow-up period. These data provide support for the thesis that without repeated exposure to trachoma, incident scarring is low, but other mechanisms for progression of scarring apart from ongoing transmission are operating, at least in children.

## Introduction

Active trachoma is defined as the presence of either trachomatous inflammation—follicular (TF) or trachomatous inflammation—intense (TI). It is most often observed in children and becomes less prevalent with age. The onset of trachomatous scarring (TS) involves repeated exposure to *C*. *trachomatis* and is likely due to the inflammatory response of the host, rather than the infection itself. Scarring most commonly manifests in adulthood and in regions with severe disease burden, it can be observed in childhood [1].The World Health Organization (WHO) estimates in 2019 that 142 million people live in trachoma endemic areas and remain at risk [2]. The organization has supported implementation of the SAFE strategy in pursuing the goal of trachoma elimination: *S*urgery to treat late complications, *A*ntibiotics to treat infection, *F*acial cleanliness and *E*nvironmental improvement to limit transmission. Implementation of this strategy has contributed to a decline in the prevalence of TF. However, there is evidence of incident scarring and scarring progression among adults in regions with low prevalence of trachoma [1]. There are few studies that have examined the incidence and progression of trachomatous scarring in children [3–5].

In a hyper-endemic area, the 5-year risk of incident scarring in children was almost six-fold higher in children with constant, severe trachoma or infection compared to those with no trachoma [6]. The time gap between repeated episodes of infection/inflammation in childhood and their sequelae in later life has led to several investigations of the role of inflammation in trachomatous scarring [4, 7]. There are differences in host response to infection, such as in microRNA profiles, between cases of scarring with inflammation versus scarring without inflammation [8] Data are emerging that suggest the development of a persistent inflammatory response due to previous infections may drive worsening scarring, even in the absence of re-exposure [3]. Thus, studying scarring in children where trachoma prevalence has become low, has the potential to help elucidate mechanisms for incidence and progression. We hypothesized that in this setting, incident scarring would be rare, but progression rates would be similar to that observed in trachoma hyper-endemic settings.

The primary objective of this study is to determine the incidence and progression of trachomatous scarring in a cohort of children residing in the formerly hyper-endemic district of Kongwa, Tanzania where the prevalence of TF was <5%. Secondary objectives include assessment of scarring severity and of variables associated with the incidence and progression of scarring.

## Methods

### Ethical statement

This study was approved by the Ethics Committee of the Tanzania National Institute for Medical Research and by the Johns Hopkins Institutional Review Board. It fully complied with the precepts of the declaration of Helsinki. Community leaders at each village provided verbal consent permitting community participation in the study. Parents or legal guardians of all participants provided informed written consent. English consent forms were translated into Swahili. Study personnel solicited informed consent in Swahili, or in the local languages of Kigogo or Kikaguru if necessary.

### Population

Kongwa is a rural district in the centrally located Dodoma region of Tanzania. The area was once a known hotspot for hyper-endemic trachoma, and a survey in 1986 noted active trachoma in over 60% of pre-school children [9]. A survey conducted in Kongwa district in 2008 found that the prevalence of active trachoma among children ages 0–5 years was 32.3% [10]. The district has since been the site of trachoma control interventions based on the WHO SAFE strategy, including mass drug administration of antibiotics [11]. By 2016, the prevalence of TF in children ages 1–9 years in the district had fallen to about 5% [11].

**Fig 1:**
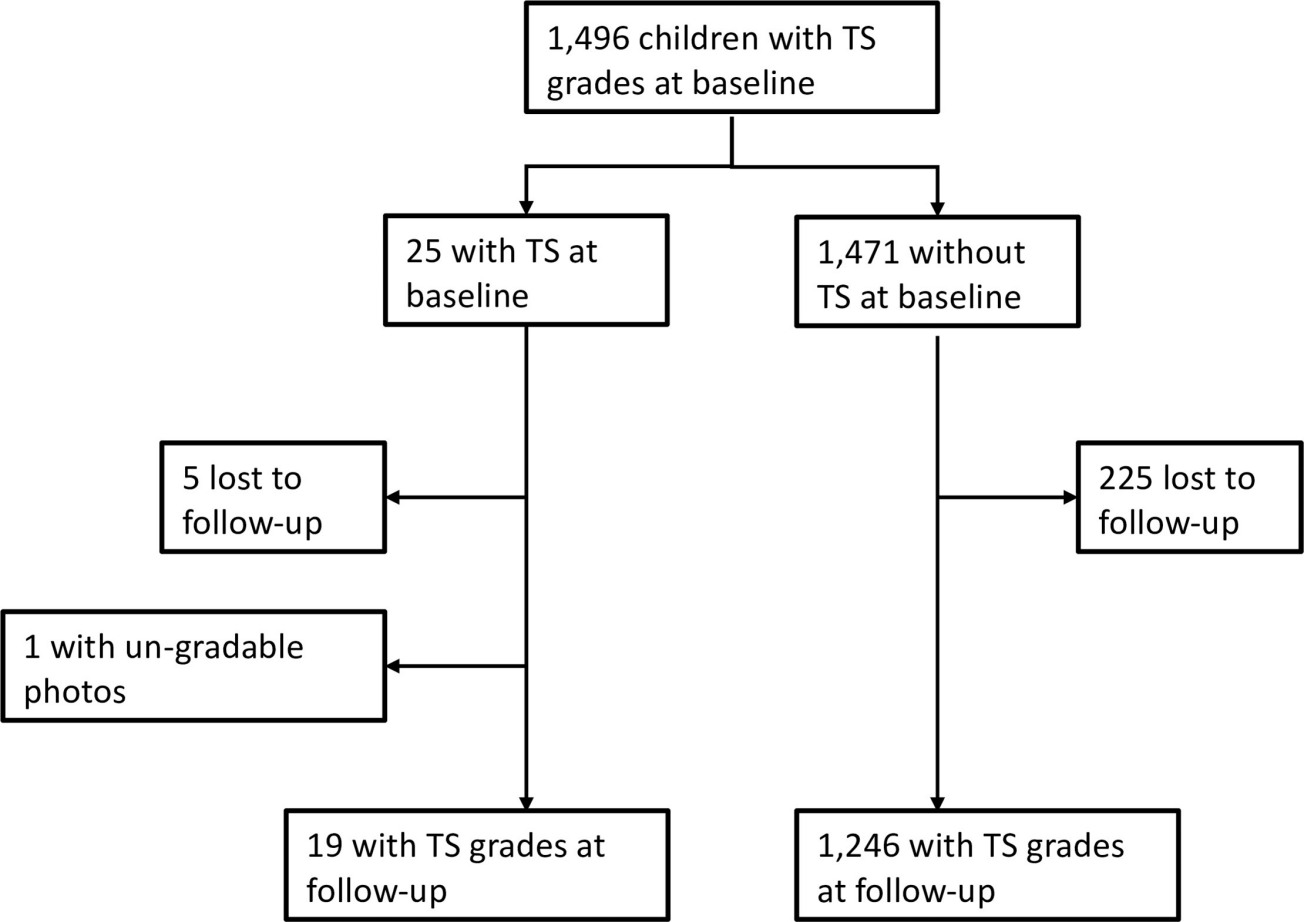
Flowchart describing participant follow-up at 2 years.

At baseline, 1920 participants ages 1–9 years were randomly selected from a census conducted in 38 villages in Kongwa in 2015. These communities were already involved in a separate study [11]. In each village, 50 children and two substitutes were randomly selected using a table of random numbers, in order to try and ensure the goal of recruiting 50 children in each community. Of the 1920, 78% (n = 1505) participated at baseline and photos were gradable for 1496. These 1496 formed the cohort for the analyses of this longitudinal study (Fig 1). Details are described in a prior publication [12].

### Study design

At baseline, a trained grader performed an ocular examination of the right upper tarsal conjunctiva using a 2.5× loupe and flashlight to evaluate the presence of TF. The grader also collected an eye swab using a Dacron swab from the child’s right eye, using single-use gloves and adherence to field protocols to avoid contamination. The swab was inserted into a sterile tube, capped by the lab technician and labeled with a code. The sample was evaluated for presence of *C*. *trachomatis* infection using the APTIMA Combo 2 assay. A 5% sample of “air swabs” was collected to evaluate potential contamination. Graders captured digital photographs of the right upper tarsal conjunctiva of each child (D40; Nikon, Tokyo, Japan). At 2-year follow-up, clinical examination, swabs for detection of *C*. *trachomatis* and tarsal photographs were repeated using exactly the same methods. At follow-up, both the left and right tarsal conjunctiva were photographed.

At both time points, photographs were graded for severity of scarring using a four-point scale developed by Wolle et al. [13] and used in several previous publications to describe scarring in children and adults [6, 12–15]. The scale ranges between S1-S4 and is detailed below (Table 1).

**Table 1:** Trachomatous scarring grading system.

Scarring Grade	Description
S1	One or more lines of scarring at least 3 mm in length, with scarring not covering 1/8 of the eyelid
S2	Multiple lines or patches of scarring, covering at least 1/8 of the eyelid conjunctiva but less than 1/3 of the eyelid
S3	Scarring covering at least 1/3 of the eyelid conjunctiva but less than 90% of the eyelid
S4	Scarring covering at least 90% of the eyelid conjunctiva

Two graders at Johns Hopkins University were trained on this method and tested for interobserver reliability using a training set of 60 images. The pair demonstrated good interrater reliability (kappa > 0.70). All photographs at baseline and follow-up were graded by each grader independently from one another and from knowledge of the baseline status of the eye. Discrepancies were adjudicated between graders and, in cases of persistent disagreement, through discussion with a senior grader.

### Analysis

The sample size was fixed by the baseline cohort. We expected an incident rate of 3%, based on prior work, and with a sample of 1246, we have over 95% confidence in a 1% level of absolute precision [16].

Descriptive statistics were used to assess differences between participants and non-participants at baseline. Due to the low rate of incidence and the small number at risk of progression of scarring during the follow-up period, all scarring grading data were analyzed as follows: incidence was defined as any scarring (S1-S4) at follow-up in individuals without scarring at baseline. Progression was defined as any worsening of scarring, by a change of at least one grade, in individuals found to have scarring at baseline. Risks of scarring incidence and progression were calculated per year. Poisson regression models were used to determine risk factors for incident scarring.

Contingency tables were used to evaluate differences between participants examined at 2-year follow-up and those not examined. Chi-square and Fischer’s exact testing were used to evaluate differences between the two groups across age, sex, *C*. *trachomatis* infection status, trachomatous scarring at baseline, community TF prevalence and several environmental and socio-economic variables (household education, latrine access, proximity to potable water and bicycle ownership). The same statistical tests and variables were evaluated in comparing those who did and did not have incident scarring at follow-up.

Potential predictors of incident scarring were incorporated into three logistic regression models–age-adjusted, multivariate and parsimonious. The multivariate model included age and risk factors associated with incidence with a p-value < 0.2. The parsimonious model was created using a backward elimination approach, retaining only the two factors with the strongest level of significance. Intra-class correlation coefficients (ICC) were used to evaluate community-based clustering of incident scarring. At follow-up, ICC were also used to assess whether scarring clusters across fellow eyes in a given participant. All analyses were conducted on SAS 9.4 software (SAS Institute, Cary, NC). Data are collated in supplementary file 1 (S1 Data). The completed STROBE checklist is presented in supplementary file 2 (S2 Data).

## Results

At baseline, 1,496 children comprised the study cohort, drawn from a cross sectional study that randomly selected and enrolled children from among 38 communities. A total of 1,266 participants were successfully followed-up at 2 years (85%). The primary reasons for loss to follow-up were being absent from the village at the time of examination (86%) and participant refusal (10%). The flow of participants is summarized in Fig 1. The only significant difference between those who were followed-up and those who were not was that participants at follow-up were younger (p-value < 0.001) (Table 2). No other characteristic was significantly different across the two groups.

**Table 2:** Baseline characteristics by follow-up status.

Baseline characteristics	Follow-up Status		p-value
	Examined N = 1266	Not Examined N = 230	
Age, mean ± SD	5.4 ± 2.6	6.0 ± 2.7	0.0006
Sex, n (%)			
Male	623 (49.2)	109 (47.4)	0.61
Female	643 (50.8)	121 (52.6)	
TF, n (%)			
Present	43 (3.4)	5 (2.2)	0.33
Absent	1223 (96.6)	225 (97.8)	
*C*. *trachomatis* infection, n (%)			
Present	80 (6.2)	17 (7.4)	0.54
Absent	1186 (93.7)	213 (92.6)	
Trachomatous scarring, n (%)			
Present	20 (1.6)	5 (2.2)	0.57
Absent	1246 (98.4)	225 (97.8)	
Formal education head of the household, n (%)			
None	445 (35.5)	95 (42.0)	0.06
Some	808 (64.5)	131 (58.0)	
Latrine in the household, n (%)			
No	262 (20.9)	48 (21.2)	0.92
Yes	989 (79.1)	178 (78.8)	
Lives more than 30 minutes from water, n (%)			
No	585 (46.7)	96 (42.5)	0.24
Yes	667 (53.3)	130 (57.5)	
Bicycle in household, n (%)			
No	623 (49.7)	115 (50.9)	0.75
Yes	630 (50.3)	111 (49.1)	
Lives in a community with TF prevalence >5.0%, n (%) *			
No	956 (75.5)	175 (76.1)	0.85
Yes	310 (24.5)	55 (23.9)	

* Assessed 6 months prior to baseline exam

Note: household characteristics are missing for some children

**Fig 2:**
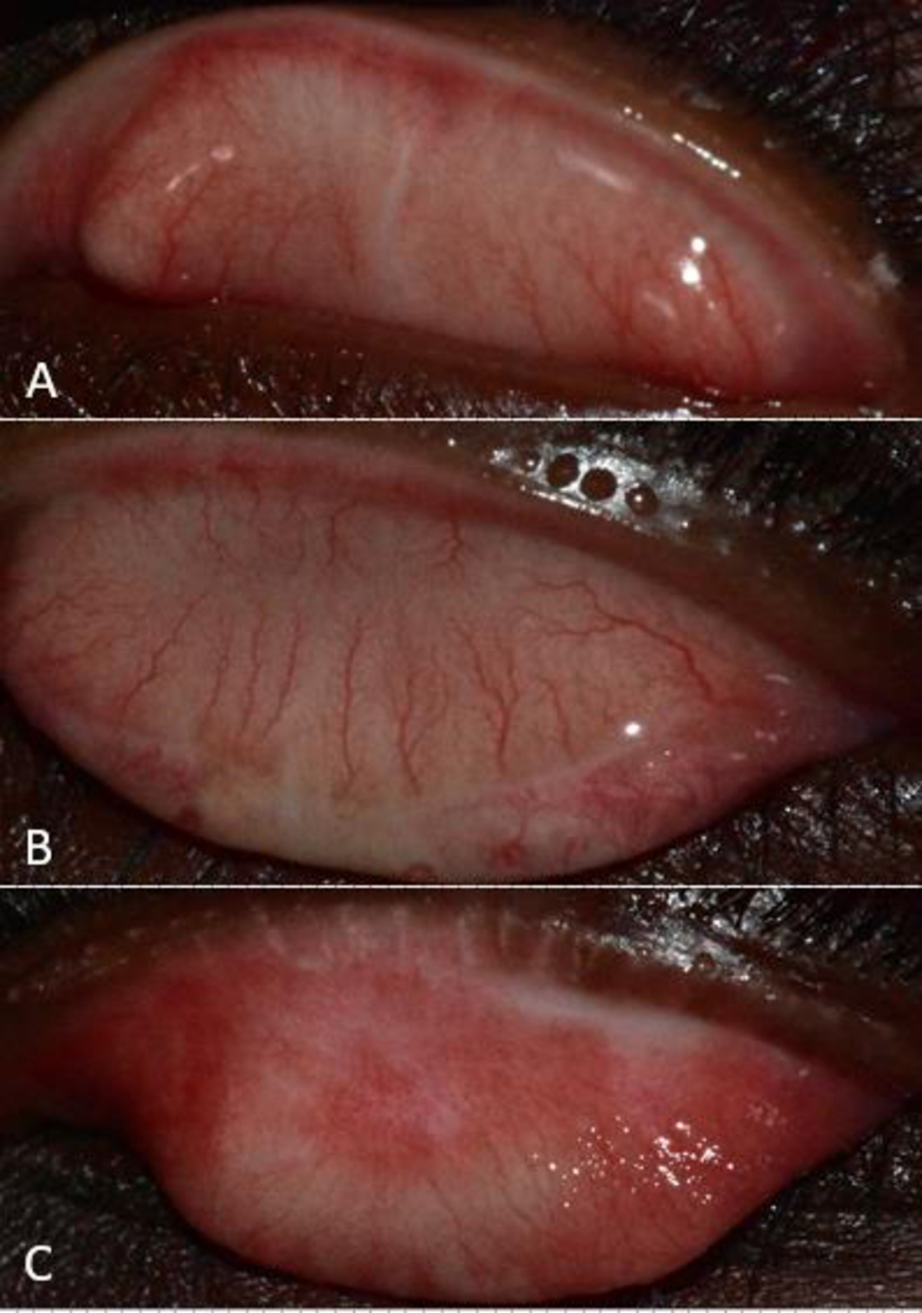
Conjunctival photographs demonstrating three grades of incident scarring in the study cohort. (A) Mild scarring (grade S1); a single linear scar is present. (B) Moderate scarring (grade S2); linear scarring spanning the width of the inner surface of the eyelid. Severe scarring (grade S3); diffuse meshwork of scarring over the conjunctival surface.

There were 20 participants with incident scarring. The 2-year incidence of scarring among at-risk participants with no scarring at baseline was 1.6% (95% CI: 0.9%-2.3%; 0.8%/year). There were 8 participants with scarring graded in the mildest category (S1), 9 with S2 scarring and 3 with S3 scarring. Those with incident scarring were more likely to be female (Table 3). There was a tendency for those with incident scarring to reside in communities that still had trachoma, but the differences were not statistically significant. Examples of incident scarring, grades S1-S3, are shown in Fig 2.

**Table 3:** Baseline characteristics by incident scarring at 2-year follow-up.

Baseline characteristics	Incident Scarring		p-value
	Absent N = 1226	Present N = 20 (2-year incident rate %)	
Age, mean ± SD	5.4 ± 2.6	5.0 ± 2.3	0.55
Sex, n			
Male	606	5 (0.82)	0.04
Female	620	15 (2.36)	
TF, n			
Present	42	1 (2.33)	0.51
Absent	1184	19 (1.57)	
*C*. *trachomatis* infection, n			
Present	78	2 (2.50)	0.37
Absent	1148	18 (1.54)	
Formal education head of the household, n			
None	432	5 (1.14)	0.48
Some	781	15 (1.88)	
Latrine in the household, n			
No	250	7 (2.72)	0.16
Yes	961	13 (1.33)	
Lives more than 30 minutes from water, n			
No	572	6 (1.04)	0.17
Yes	640	14 (2.14)	
Bicycle in household, n			
No	600	12 (1.96)	0.38
Yes	613	8 (1.29)	
Lives in a community with TF prevalence >5.0%, n *			
No	930	12 (1.27)	0.12
Yes	296	8 (2.63)	

* Assessed 6 months prior to baseline exam

Note: household characteristics are missing for some children

The potential association of risk factors with incident scarring was explored in three types of Poisson regression models (Table 4). The first set of models only adjusted for age. The multivariate model included: age, sex, latrine access, distance from water, and community TF prevalence >5%. The final parsimonious model included sex and community TF prevalence >5%. Across all models, incidence of scarring was associated with female sex (Multivariate model RR = 2.92 (95% CI = 1.06–8.04; p-value < 0.05). There were trends toward an association between incidence and distance from water sources and with community TF prevalence >5%. There was also a trend toward a protective effect of household latrine access. None reached statistical significance (Table 4). There was no evidence of clustering of incident cases across included communities (ICC = 0.05; 95% CI = -0.23–0.32).

**Table 4:** Models predicting incidence of scarring.

Characteristic	Incidence rate ratio (95% CI), p-value		
	Age-adjusted	Multivariate Model	Parsimonious Model
Female	2.93 (1.06–8.06), 0.04	2.92 (1.06–8.04), 0.04	2.93 (1.06–8.06), 0.04
Latrine in household	0.50 (0.20–1.25), 0.14	0.59 (0.23–1.52), 0.27	_________________
More than 30 minutes from water source	2.06 (0.79–5.37), 0.14	1.63 (0.60–4.41), 0.34	_________________
Lives in a community with estimated TF prevalence >5%	2.04 (0.83–5.00), 0.12	1.79 (0.72–4.45), 0.21	2.04 (0.83–5.00), 0.12

Twenty participants with prevalent scarring at baseline were successfully followed-up at 2 years (80%). Of these, 1 participant had un-gradable photos of the tarsal conjunctiva and was excluded from our analysis. Among those followed-up and with images, 21% (4/19) showed progression of scarring. This amounts to an annual risk of progression of 10.5%/year. All cases of progression of scarring were in female children. From the images obtained at follow-up, we examined the interrelationship of scarring in right and left eyes and found evidence of correlation between eyes (ICC = 0.971; 95% CI = 0.950–0.992; p < 0.0001).

## Discussion

In this cohort study, we examined the 2-year incidence and progression of scarring among children in a formerly trachoma hyper-endemic district of Central Tanzania. There was high rate of follow-up of the cohort at baseline, at 85%. Participants lost to follow-up were older. This is likely due to the tendency for older children to engage in work or formal education away from the household.

The period incidence of scarring in this sample was low, at 1.6% (estimate of 0.8%/year). This incidence rate was calculated using right eye photographs only because that was what was available at baseline. Even with a high correlation between eyes, the incident rate per person-year may be higher if there were incident cases of left eye only. We attempted to estimate the size of this issue as follows: at follow-up, there were 9 cases of trachomatous scarring in left eyes of participants who had no right eye scarring, but we cannot determine if they are all incident scars or some were already present at baseline. It is impossible to determine whether these cases of left eye scarring were incident or prevalent. However, 51% of the right eyes at follow up without scarring in the left eye were incident cases. Assuming a similar rate among left eyes, we can surmise that about 5 of these 9 cases of left eye scarring were incident cases, resulting in an estimated scarring incidence rate of about 2.0%, or around 1% per year. This estimate is still below that estimated in children of the same age in Kongwa from a time of higher trachoma endemicity [6]. In that study, children with constant, severe trachoma or infection had an annual incident rate of scarring between 6–9%; those with occasional TF or infection had an annual rate of 3%. Overall, the annual incident rate was 3.8% in children ages 1–10 years [13].

A recent four-year cohort study of children ages 6–10 years in another district in Tanzania reported an incidence rate of 2.7%/year [4]. The findings of this latter study are not comparable to ours for several reasons. The children in the study were 5 years older at baseline and the study area had a higher baseline prevalence of active trachoma. The utilized grading scheme differed from our system and had a lower threshold for the lowest grade of scarring [5]. Moreover, grading of conjunctival photographs for incidence and progression was conducted as side by side comparison. This may have permitted graders to better detect changes, at the expense of potentially biasing grading.

The low incident rate coupled with the distribution of graded severity of incident scarring is noteworthy. We found that 60% (12/20) had incident scarring in the moderate and severe grading tiers (S2 and S3). This suggests that there is a small cohort of children with rapid development and progression of trachomatous scarring. Moreover, this disease course was observed in the setting of low prevalence of active trachoma. To place this observation in context, let us say there are at least two different scenarios in which scarring incidence and progression from trachoma can occur: one in a hyper-endemic setting, in which transmission is ongoing and relatively intense. The other setting is where trachoma was once hyper-endemic but is now hypo-endemic. In a hyper-endemic setting, we see incident scarring related to constant severe trachoma and infection, as reported previously by our group [3, 6]. In such a setting, it is difficult to determine how much of the scarring is due to ongoing transmission and multiple bouts of infection leading to incident scarring, and if there is a select group with a predisposition (genetic or epigenetic) to mount a florid inflammatory response to just a few bouts of infection leading to scarring. Differential host responses to *C*. *trachomatis* infection is not a new concept [7]. It is also known that there are different patterns of scarring associated with differences in expression of factors like host microRNA between scarring with inflammation and scarring without inflammation [8]. In a hypo-endemic setting, though, community transmission intensity is greatly reduced, and one would expect much reduced incidence of scarring. This indeed was what was observed in our current study, where incident scarring has decreased in children by almost a third compared to children in Kongwa when trachoma was hyper-endemic [6]. However, in the current study, more than half of the incident scarring cases progressed relatively rapidly over the period to grade 2 and 3 scarring. By removing the intensity of transmission, since trachoma was hypo-endemic over the study period, we argue that we may have revealed more clearly a small subset of children who are predisposed to scarring and progression after few exposures. In studies of adults, the prevalence of trachomatous scarring (TS) is known to increase with age. However, at baseline, we had found that the prevalence of scarring in our cohort of children ages 1–9 years did not increase with age [12]. We now find that incidence also did not increase with age. This is similar to work by Ramadhani et al., where incidence and progression did not increase significantly with age [4]. In prior work, we had seen a difference by age in prevalence of scarring in a hyper-endemic area in Kongwa district. In that study, children ages 1-5-years had a 1% prevalence of TS, compared to 12% in children ages 6–10 years [13]. Incidence rates in those two age groups also showed a slight difference, with a higher rate in the older cohort. It may be that in a hyper-endemic setting, where everyone is exposed, the risk of scarring reflects age as a surrogate of number of exposures; in a hypo-endemic setting, the risk depends on immediate family setting and other factors which are more independent of age.

Incidence of scarring was associated with female sex. The association persisted even after adjusting for covariates. This finding has been repeatedly demonstrated in prior studies, including in children [3, 6, 13]. It has been hypothesized that the relationship as observed in adults is driven by greater female exposure to children. Our findings are not necessarily inconsistent with this hypothesis, as we have observed girls in these communities carrying their younger siblings. However, because trachoma prevalence is so low in this setting, it is unclear that this behavior substantially increases their exposure to repeated bouts of infection.

The presence of this association in young children strengthens the possibility that sex-based factors may also be operative. The corneal epithelium is known to be estrogen sensitive, so it is tempting to make analogies with the female genital tract and risk of genital Chlamydia. For example, work by Davis et al. shows that a constituent of the estrogen receptor complex on the host cell enhances Chlamydial infectivity in an endometrial cell line model [17]. The conjunctival epithelium appears to be estrogen sensitive [18] and it may be reasonable to hypothesize that girls may have enhanced infectivity once exposed to Chlamydia. However, it is unclear whether these receptors are differentially distributed across sex, particularly in our pre-pubescent cohort. Moreover, it has been difficult to demonstrate estrogen receptors in epithelium [19, 20].

In addition to possible differential host/infection interactions, one might speculate about sex- differences in immune response. Women are known to have higher rates of a wide range of autoimmune conditions, some of which precede the onset of puberty [21, 22]. If TS is viewed as the product of a disordered immune inflammatory response to *C*. *trachomatis* infection, it may be reasonable to hypothesize an association with female sex.

Among those who had scarring at baseline, the rate of progression was 10.5%/year. This finding is consistent with the extant literature. Our previous prospective cohort study conducted in a hyper-endemic area in Kongwa reported a TS progression rate of about 12.7%/year in a sub-group of participants ages 1–5 years at baseline [13]. The progression occurred regardless of the fact that the overall TF rate in this district was <5%. This is consistent with findings that suggest the progression of scarring ensues because of a cascade that is able to operate independent of *C*. *trachomatis* re-infection. Ramadhani et al., found that inflammation is a stronger predictor of scarring progression, and that progression was not associated with infection [4, 23].

A number of limitations merit consideration. First, incidence and progression were based on findings in one eye. In general, there is a strong correlation between eyes, and we had an ICC between fellow eyes of 0.97 at follow-up, so we do not feel there is significant bias in assessing incidence or progression. Second, low rates of incident scarring and low numbers at risk of progression meant that some associations were under-powered. For example, incidence may be related to residence in a village with TF prevalence of 5% or greater, but we could not find the association statistically significant, in part due to the small numbers of children resident in villages that still have trachoma. We found that all four cases of progression of scarring were in female children. However, limited statistical power meant that we were not able to pursue this, and other variables potentially associated with progression. Third, we are not able to ascertain that all incidence and progression of scarring was due to trachoma, a limitation of all scarring studies as there are non-chlamydial causes of conjunctival scarring such as trauma and other bacterial and viral infections [24].

In conclusion, the 2-year incidence of scarring among children in these formerly hyper-endemic communities in Central Tanzania was low, at 1.6%. Incidence was higher in females. In children who had prevalent scarring at baseline, the rate of scarring progression was 21% (10.5%/year). This highlights the importance of primary prevention of scarring through the SAFE strategy, and suggests long-term surveillance of individuals with scarring, even in hypo-endemic areas, may be necessary to determine ongoing risk of trichiasis.

## Supporting information

S1 Data Excel spreadsheet with raw data. (XLSX)

S2 Data STROBE checklist outlining compliance with STROBE criteria for cohort studies. (DOC)
